# Neutrophil‐Lymphocyte Ratio as Predictor for Acute Infection After Primary Total Joint Arthroplasty in Rheumatoid Arthritis Patients

**DOI:** 10.1111/os.70002

**Published:** 2025-02-26

**Authors:** Yahao Lai, Jiaxuan Fan, Ning Lv, Xiaoyu Li, Wenxuan Zhao, Zeyu Luo, Zongke Zhou

**Affiliations:** ^1^ Department of Orthopaedic Surgery West China Hospital, Sichuan University Chengdu China; ^2^ West China School of Public Health and West China Fourth Hospital, Sichuan University Chengdu China; ^3^ Department of Pharmacy State Key Laboratory of Biotherapy, Sichuan University Chengdu China

**Keywords:** arthroplasty, infection, inflammatory biomarkers, neutrophil‐lymphocyte ratio, rheumatoid arthritis

## Abstract

**Objectives:**

Preoperative levels of certain inflammatory markers in the blood can predict acute infection after primary total joint arthroplasty in patients without inflammatory disease, but whether they can do so in patients with rheumatoid arthritis is unclear. The objectives of this study were to determine whether, with appropriate cut‐off values, (1) preoperative levels of NLR predicted postoperative acute infection; and (2) preoperative plasma fibrinogen, monocyte‐lymphocyte ratio, C‐reactive protein or erythrocyte sedimentation rate predicted postoperative acute infection.

**Methods:**

We retrospectively analyzed 964 patients with rheumatoid arthritis who underwent primary total joint arthroplasty at our hospital between January 2010 and November 2020. We compared preoperative levels of inflammatory markers including neutrophil‐lymphocyte ratio (NLR), monocyte‐lymphocyte ratio (MLR), C‐reactive protein (CRP), erythrocyte sedimentation rate (ESR), plasma fibrinogen (FIB) between patients who suffered acute infection or not within 90 days after surgery. The ability of markers to predict infection was assessed in terms of the area under receiver operating characteristic curves (AUC) based on optimal cut‐off values determined from the Youden index.

**Results:**

Among the 964 patients, 27 (2.8%) experienced acute infection. Preoperative levels of individual inflammatory markers predicted infection with the following AUCs and cut‐off values: NLR, 0.704 (cut‐off: 2.528); MLR, 0.608 (0.2317); CRP, 0.516 (4.125 mg/L); ESR, 0.533 (66.5 mm/h); and FIB, 0.552 (3.415 g/L). The neutrophil‐lymphocyte ratio showed diagnostic sensitivity of 92.6% and specificity of 43.3%, while the monocyte‐lymphocyte ratio showed sensitivity of 77.8% and specificity of 46.3%.

**Conclusion:**

The preoperative NLR shows some ability to predict acute infection after total joint arthroplasty in patients with rheumatoid arthritis. Monitoring this ratio, perhaps in conjunction with other markers not analyzed here, may be useful for optimizing the timing of surgery in order to minimize risk of postoperative infection.

## Graphical abstract

Receiver operating characteristic curves to assess the ability of preoperative levels of inflammatory markers to predict acute infection within 90 days after total joint arthroplasty in patients with rheumatoid arthritis. (A) C‐reactive protein (CRP), erythrocyte sedimentation rate (ESR), or plasma fibrinogen (FIB). (B) Neutrophil‐lymphocyte ratio (NLR) or monocyte‐lymphocyte ratio (MLR). The red line represents perfect performance. NLR showed the best predictive value.

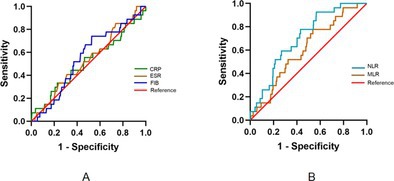

## 1 Introduction

Rheumatoid arthritis is an autoimmune process of unknown etiology involving systemic inflammation that leads to joint pain, swelling, stiffness, severe deformity and loss of function [1]. Total joint arthroplasty is widely used to restore lost function and relieve pain in patients with end‐stage rheumatoid arthritis [2] and, although it is often effective [3], many patients experience surgical complications [4, 5] such as periprosthetic joint infection and superficial infection, reflecting the use of immunosuppressive drugs [6]. In fact, infections in the mouth or elsewhere in the body can increase the risk of surgical site infection [7]. Such infections can be difficult to diagnose and treat, substantially increasing medical costs [8]. In addition, screening for acute infection risk allows for earlier control of potential infections, and potential implant retention procedure can be recommended before conversion to chronic infection [9]. Reliable preoperative biomarkers to predict the risk of acute infection after total joint arthroplasty could help personalize treatment and postoperative management, increasing the efficiency of medical resource use.

Several blood indices that are measured before primary surgery have proven effective for predicting periprosthetic joint infection after total joint arthroplasty in patients with non‐inflammatory arthritis [10, 11, 12]. Similarly, some studies have shown that these blood biomarkers measured after primary surgery can be used to diagnose potential acute infections in patients with non‐inflammatory arthritis [13, 14]. These indices include C‐reactive protein, erythrocyte sedimentation rate, plasma fibrinogen, monocyte‐lymphocyte ratio, and neutrophil‐lymphocyte ratio (NLR). Among these, NLR has consistently been linked to inflammation and infection in a variety of contexts [15, 16] and to mortality in patients with pulmonary Infection [17]. Whether NLR or other blood indices can reliably predict postoperative injection in patients with an inflammatory disease such as rheumatoid arthritis is unclear: the higher baseline levels of inflammatory markers in the blood of such patients may “mask” any increases linked to acute infection [18]. At the very least, the cut‐off values for predicting infection would probably need to be higher than those in previously reported cut‐off values.

We explored these questions in a relatively large sample of patients with rheumatoid arthritis at our medical center who underwent total joint arthroplasty. We retrospectively analyzed whether, with appropriate cut‐off values, (1) preoperative levels of NLR predicted postoperative acute infection; and (2) preoperative plasma fibrinogen, monocyte‐lymphocyte ratio, C‐reactive protein or erythrocyte sedimentation rate predicted postoperative acute infection.

## 2 Methods

### 2.1 Patients

We retrospectively analyzed a consecutive series of patients with end‐stage rheumatoid arthritis who underwent total knee or hip arthroplasty at our large teaching hospital between January 2010 and December 2020. Patients had to be (1) at least 18 years old but no older than 80 years, (2) diagnosed with rheumatoid arthritis based on the criteria of the American College of Rheumatology and European League Against Rheumatism [19], and (3) underwent primary total knee or hip arthroplasty. Patients were excluded if they (1) were lost to follow‐up within 90 days after surgery; (2) missed laboratory data.

This retrospective study was approved by Ethics Committee on Biomedical Research of West China Hospital of Sichuan University (approval 2023–2005), which waived the requirement for informed consent because patients, at the time of treatment, consented for their anonymized data to be analyzed and published for research purposes.

All included patients were retrieved through the electronic medical record system and had complete medical records. We retrospectively collected patients baseline data, including sex, age, BMI, comorbidities, type of surgery, American Society of Anesthesiologists (ASA) grade, history of medication for rheumatoid arthritis, and diabetes mellitus. In addition, inflammatory markers on the day of admission including CRP, ESR, FIB, neutrophil, lymphocyte, and monocyte counts were collected.

The same perioperative management protocols were used for all patients, including blood loss reduction, infection prevention, pain control blood glucose control, and postoperative rehabilitation training. For patients with rheumatoid arthritis, conventional disease‐modifying anti‐rheumatic drug (DMARDS) were allowed to continue before surgery to reduce postoperative complication rates, whereas all biologic medications were discontinued for one cycle before surgery. All arthroplasties in this study were conducted using standard procedures under the same analgesic protocol. All patients received antibiotics during the perioperative period to prevent infection. Selective COX‐2 inhibitors have been used for pain relief. All arthroplasties were performed by five experienced joint surgeons using a standardized surgical approach to ensure similar surgical trauma among all patients. Blood glucose was monitored daily during hospitalization. Patients with poorly controlled blood glucose at discharge were advised to self‐monitor regularly.

The outpatient clinic visits were scheduled for all patients at 1 and 3 months post‐discharge. During each follow‐up appointment, comprehensive physical examinations and x‐ray assessments were conducted to detect any potential postoperative complications.

### 2.2 Diagnosis of Postoperative Acute Infection

Patients were assigned to two groups depending on whether they experienced acute infection, diagnosed according to published criteria [20], within 90 days after total joint arthroplasty [14].

At discharge from hospital after arthroplasty, patients were asked to return to our hospital immediately if they felt abnormal pain, swelling, or discharge of pus from the wound. If infection was suspected based on symptoms, imaging, and preoperative laboratory results, the affected joint was aspirated and synovial fluid was cultured for bacteria. In addition, at least four periprosthetic soft tissues were analyzed by histopathology and cultured for bacteria. All culture and histopathology procedures were performed in‐hospital.

All patients in the study were followed up either until diagnosis with postoperative acute infection or until 90 days after arthroplasty, whichever occurred first. Patients who did not return to the hospital because of infection were followed up during outpatient visits in order to confirm the absence of infection at 90 days.

### 2.3 Preoperative Assay of Inflammatory Markers

On the day before total joint arthroplasty, blood was sampled and analyzed for level of C‐reactive protein in serum, erythryocyte sedimentation rate, plasma fibrinogen, and counts of neutrophils, lymphocytes and monocytes. Blood samples were analyzed in‐hospital within 2 h of collection.

### 2.4 Data Extraction and Outcomes

Data were collected on the following clinicodemographic characteristics of patients: sex, age, body mass index, preoperative diagnosis, preoperative comorbidities such as diabetes, Charlson comorbidity index, type of joint involved in the surgery, and American Society of Anesthesiologists grade.

Data were also collected on the following outcomes: preoperative values of C‐reactive protein level, erythrocyte sedimentation rate, plasma fibrinogen, monocyte‐lymphocyte ratio, and neutrophil‐lymphocyte ratio (NLR), and 90‐day rate of acute infections (superficial or periprosthetic joint infection).

### 2.5 Statistical Analyses

All statistical analyses were performed using SPSS 26.0 (IBM, Armonk, NY, USA) and GraphPad Prism 9.0 for Windows (GraphPad, Boston, MA, USA), and results associated with *p* < 0.05 were considered significant. Continuous data such as age, body mass index were reported as mean ± standard deviation (SD), and intergroup differences were assessed for significance using the independent‐samples *t* test or Mann–Whitney *U* test. The Mann–Whitney *U* test was used to assess differences in preoperative levels of inflammatory markers, including C‐reactive protein level, erythrocyte sedimentation rate, plasma fibrinogen, monocyte‐lymphocyte ratio, and neutrophil‐lymphocyte ratio. Categorical data such as Charlson comorbidity index, type of joint being operated, American Society of Anesthesiologists grade, Medication history, and Diabetes were reported as *n* (%), and intergroup differences were assessed using the Pearson chi‐squared test, Yates's correction for continuity or Fisher's exact test, as appropriate.

The ability of preoperative levels of inflammatory markers to predict acute infection within 90 days after surgery was assessed in terms of the area under receiver operating characteristic curves (AUC) based on optimal predictive cut‐off values according to the Youden index. AUCs were reported together with their 95% confidence interval (CI). Sensitivity, specificity, positive predictive value, and negative predictive value were also calculated.

## 3 Results

### 3.1 Patients

**FIGURE 1:**
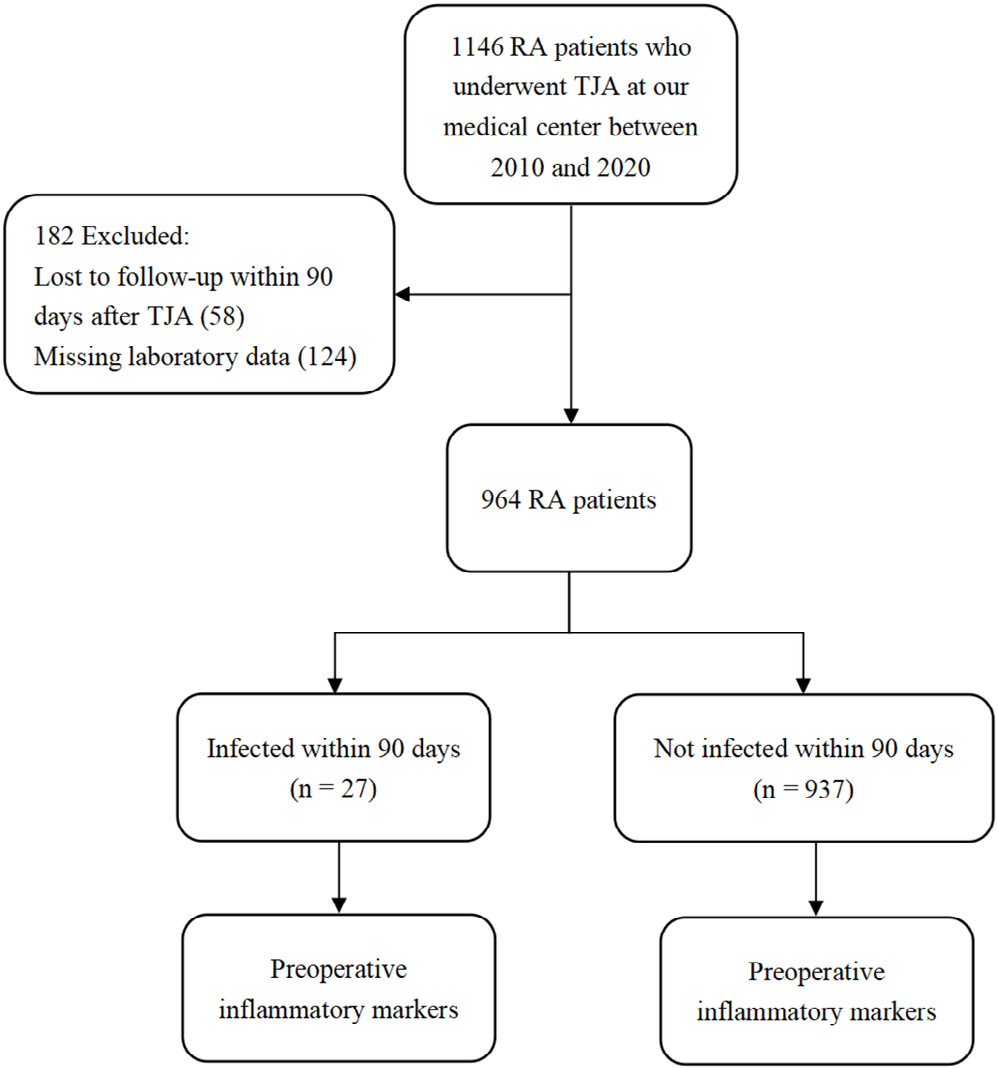
Flowchart of patient enrollment and analysis. RA, rheumatoid arthritis; TJA, total joint arthroplasty.

Of the 1146 patients whom we screened for enrollment, we excluded 58 because they were lost to follow‐up and 124 because preoperative values for one or more inflammatory markers were missing (Figure 1). In the end, we analyzed 964 patients, 27 (2.8%) of whom experienced acute infection within 90 days after surgery. Of the 27 infections, of which 10 patients (3 hips, 7 knees) experienced superficial infection and 17 patients (8 hips, 9 knees) experienced periprosthetic joint infections. The two groups of patients who experienced infection or not did not differ significantly in sex distribution, age, body mass index, Charlson comorbidity index, type of joint involved in the surgery, American Society of Anesthesiologists grade, or prevalence of diabetes (Table 1).

**TABLE 1:** Clinicodemographic characteristics of patients, stratified by acute infection within 90 days after surgery.

Characteristic	No infection (*n* = 937)	Infection (*n* = 27)	*t* or *χ* ^2^ value	*p* ^a^
Sex			—	0.117
Male	160 (17.1)	8 (29.6)		
Female	777 (82.9)	19 (70.4)		
Age, years	54.9 ± 11.7	52.9 ± 13.3	0.872	0.383
Body mass index, kg/m^2^	22.4 ± 3.7	22.5 ± 3.7	−0.138	0.890
Charlson comorbidity index			0.424	0.515
< 2	476 (50.8)	12 (44.4)		
≥ 2	461 (49.2)	15 (55.6)		
Type of joint being operated			0.576	0.448
Hip	316 (33.7)	11 (40.7)		
Knee	621 (66.3)	16 (59.3)		
American Society of Anesthesiologists grade			—	0.785
< 3	796 (85.0)	24 (88.9)		
≥ 3	141 (15.0)	3 (11.1)		
Medication history			1.605	0.448
Glucocorticoids only	117 (12.5)	7 (25.9)		
DMARDs only	138 (14.7)	4 (14.8)		
Both	119 (12.7)	4 (14.8)		
Diabetes			—	1.000
No	868 (92.6)	25 (92.6)		
Yes	69 (7.4)	2 (7.4)		

*Note:* Values are *n* (%) or mean ± SD, unless otherwise noted. “—” represents fisher's exact calculation method, *χ* ^2^ value cannot be calculated.

Abbreviation: DMARD, disease‐modifying anti‐rheumatic drug.

^a^ Based on Student's *t*‐test (continuous variables) and Person *χ* ^2^ test (categorical variables) or Fisher's exact test (categorical variables).

### 3.2 Predictive Value of Preoperative Laboratory Tests

**FIGURE 2:**
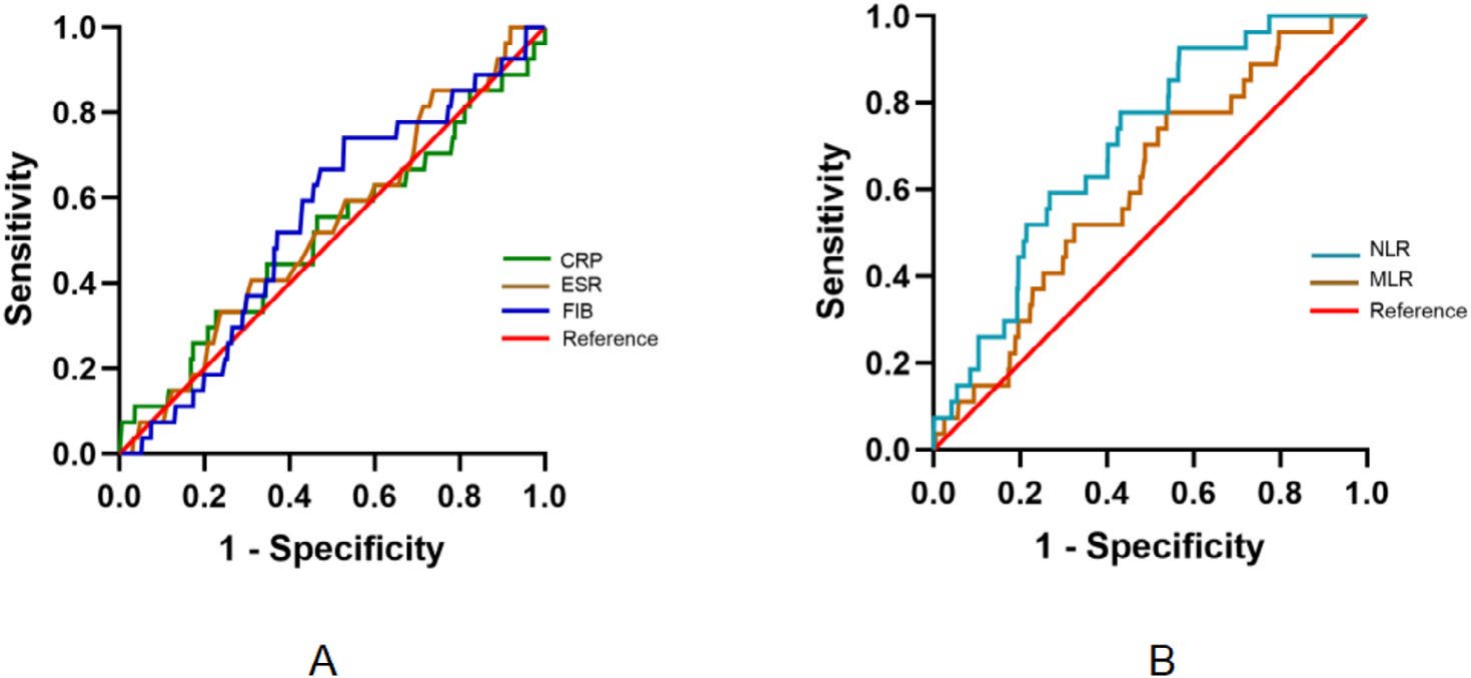
Receiver operating characteristic curves to assess the ability of preoperative levels of inflammatory markers to predict acute infection within 90 days after total joint arthroplasty in patients with rheumatoid arthritis. (A) C‐reactive protein (CRP), erythrocyte sedimentation rate (ESR), or plasma fibrinogen (FIB). (B) Neutrophil‐lymphocyte ratio (NLR) or monocyte‐lymphocyte ratio (MLR). The red line represents perfect performance.

The infection group showed significantly higher NLR (4.58 [3.16–6.12] vs. 2.77 [1.93–4.24], *p* < 0.001) before surgery than the non‐infection group, but otherwise the two groups did not differ significantly in any of the other inflammatory markers that we measured (Table 2). Consistently, we found that only NLR gave an AUC > 0.7, where 0.7 is considered the threshold of acceptable diagnostic performance [20], whereas the other markers gave AUCs below 0.7 (Figure 2; Table 3). NLR was the best predictor of acute infection after total joint arthroplasty (cutoff 2.528) (AUC: 0.704 [95% CI 0.621–0.787]). The remaining inflammatory markers all showed unsatisfactory predictive results. The AUC of CRP, ESR, FIB, and MLR was 0.516 (95% CI 0.395–0.637), 0.533 (95% CI 0.426–0.640), 0.552 (95% CI 0.451–0.653), and 0.608 (95% CI 0.511–0.706), respectively (Table 3). The cutoff of CRP, ESR, FIB, and MLR was 4.125 mg/L, 66.5 mm/h, 3.415 g/L, and 0.2317 respectively. The highest sensitivity was NLR (92.6%), and the lowest was CRP (33.3%). The highest specificity was CRP (77.3%) and the lowest was FIB (21.2%).

**TABLE 2:** Preoperative levels of inflammatory markers in patients, stratified by acute infection within 90 days after surgery.

Marker	No infection (*n* = 937)	Infection (*n* = 27)	*Z* value	*p* ^a^
C‐reactive protein (mg/L)	8.79 (4.43–16.05)	8.01 (3.36–18.20)	−0.284	0.776
Erythrocyte sedimentation rate (mm/h)	46 (29–68)	43 (26–63)	−0.583	0.560
Plasma fibrinogen (g/L)	3.47 (2.83–4.17)	3.80 (3.11–4.15)	−0.920	0.358
Monocyte‐lymphocyte ratio	0.24 (0.18–0.35)	0.31 (0.23–0.39)	−1.922	0.055
Neutrophil‐lymphocyte ratio	2.77 (1.93–4.24)	4.58 (3.15–6.12)	−3.614	< 0.001

*Note:* Data are median (interquartile range), unless otherwise indicated.

^a^ Based on Mann–Whitney *U* test.

**TABLE 3:** Ability of preoperative levels of individual inflammatory markers to predict acute infection within 90 days after surgery.

Marker	AUC (95% CI)	Youden index	Optimal marker cut‐off value^a^	Sensitivity (%)	Specificity (%)	PPV (%)	NPV (%)
C‐reactive protein	0.516 (0.395–0.637)	0.1060	4.125 mg/L	33.3	77.3	2.4	95.9
Erythrocyte sedimentation rate	0.533 (0.426–0.640)	0.1138	66.5 mm/h	85.2	26.2	1.6	96.7
Plasma fibrinogen	0.552 (0.451–0.653)	0.2124	3.415 g/L	74.1	21.2	3.9	98.4
Monocyte‐lymphocyte ratio	0.608 (0.511–0.706)	0.2410	0.2317	77.8	46.3	4.0	98.6
Neutrophil‐lymphocyte ratio	0.704 (0.621–0.787)	0.3592	2.528	92.6	43.3	4.5	99.5

Abbreviations: AUC, area under receiver operating characteristic curve; CI, confidence interval; NPV, negative predictive value; PPV, positive predictive value.

^a^ Determined based on the Youden index.

### 3.3 Optimal Cutoff Values of Preoperative Laboratory Tests

**FIGURE 3:**
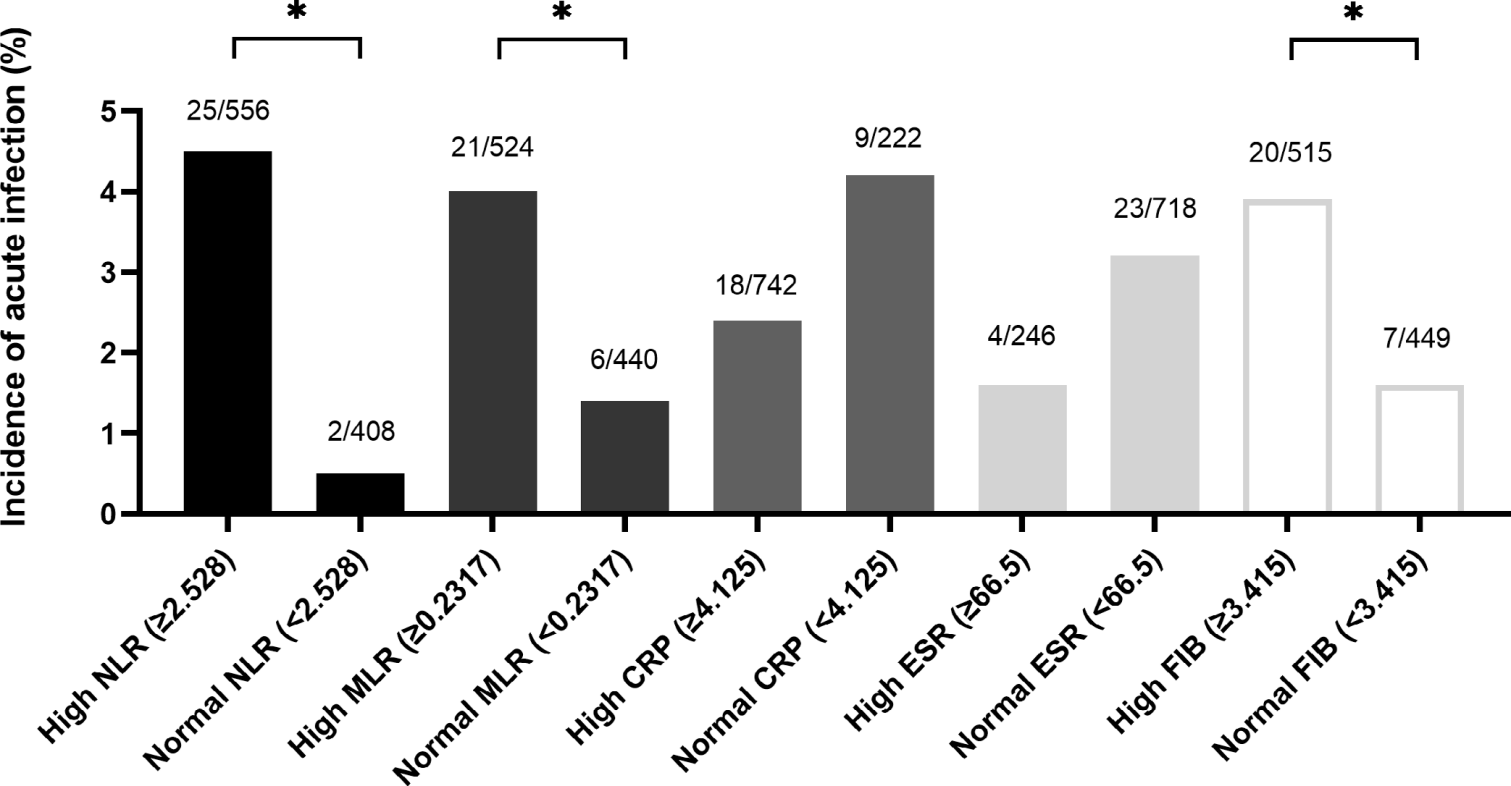
Incidence of acute infection in subgroups of patients stratified by whether their preoperative levels of inflammatory markers were below or above the optimal cut‐off according to the Youden index. Numbers of patients with infection and total patients in each subgroup (*n*/*N*) are indicated above the bars. Markers are abbreviated as in Figure 2. *p < 0.05.

When we stratified patients into “low” or “high” groups according to the optimal Youden cut‐off values, the rate of infection differed significantly between those with low or high NLR (2/408, 0.5% vs. 25/556, 4.5%, *p* < 0.001), those with low or high monocyte‐lymphocyte ratio (1.4% vs. 4.0%, *p* = 0.013) and those with low or high plasma fibrinogen (1.6% vs. 3.9%, *p* = 0.029), but not between groups stratified using the other inflammatory markers (Figure 3).

## 4 Discussion

The main finding of our study is that the preoperative NLR showed better ability to predict acute infection after total joint arthroplasty in patients with rheumatoid arthritis. This study, which appears to be the first to assess whether preoperative levels of routinely assayed inflammatory markers can predict acute infection after total joint arthroplasty in patients with rheumatoid arthritis, suggests that NLR shows borderline predictive ability (AUC 0.704, 95% CI 0.621–0.787). Further research should explore whether combining it with other markers not analyzed here would be useful for timing surgery for such patients. Levels of alpha‐defensin and leukocyte esterase [20], for example, may prove useful for this purpose.

### 4.1 Preoperative NLR Predicted Postoperative Acute Infection

Our finding with NLR extends the list of clinical contexts in which it may be a useful marker. In previous work, it helped diagnose community‐acquired pneumonia [15], and it was shown to be an independent risk factor for mortality in patients with COVID‐19 [21]. The usefulness of NLR may reflect the fact that inflammatory responses, such as those due to infection, stimulate production of neutrophils while promoting apoptosis of lymphocytes. NLR was composed of neutrophil count and lymphocyte count. Elevated neutrophil counts represent activated nonspecific inflammation and are associated with increased oxidative stress, neutrophil extracellular capture, and endothelial dysfunction [22]. Low lymphocyte counts represent poor overall health and increased physiological stress, indicating a dysregulated host immune response to surgery [23]. Considering the complex nonlinear relationship between neutrophil and lymphocyte counts, the combination of neutrophils and lymphocytes improves prognostic value over their individual components. In the particular context of rheumatoid arthritis, higher NLR may also reflect more active disease and therefore stronger systemic inflammation, consistent with the fact that neutrophils secrete pro‐inflammatory factors in the synovial environment [24]. Poor control of rheumatoid arthritis activity before surgery has been shown to increase risk of aseptic loosening [25], so it should be investigated as a risk factor for infection in future research. Since acute postoperative infection can be catastrophic to the patients, total joint arthroplasty is usually an elective procedure. If we can screen patients well before surgery by some predictive indicators, such as NLR, MLR and other indicators, we can determine the timing of surgery, and may reduce the incidence of postoperative infection. It is worth noting that although the sensitivity of NLR reached 92.6%, the specificity was not high enough. We may need to combine with other inflammatory markers to make up for this deficiency. In addition, due to the limitation of sample size, our retrospective study could not separate total joint arthroplasty into total hip arthroplasty and total knee arthroplasty subgroups, which may lead to biased results. However, considering that NLR can also reveal the preoperative systemic inflammatory response of patients, we believe that it can also be used as a feasible indicator for preoperative prediction of postoperative infection without differentiating subgroups. Of course, future studies with larger sample sizes, including detailed subgroup analyses, are needed to confirm our conclusions.

### 4.2 Preoperative C‐Reactive Protein Predicted Postoperative Acute Infection

C‐reactive protein did not predict acute infection in our patients, even though it is widely used to monitor systemic inflammation and disease activity in rheumatoid arthritis. Our negative result may reflect that C‐reactive protein is not always associated with rheumatoid arthritis activity: episodes of the disease sometimes do not involve increases in C‐reactive protein [26]. In addition, C‐reactive protein levels can decrease in response to corticosteroids and disease‐modifying anti‐rheumatic drugs [27]. It is worth noting that C‐reactive protein levels are routinely monitored before total joint arthroplasty in our center. Increases in patient's C‐reactive protein level more than three times normal usually delays surgery. This decision may have resulted in our inclusion of a smaller sample of patients with higher C‐reactive protein levels. The inclusion of follow‐up results from Rheumatoid arthritis patients with high preoperative C‐reactive protein levels in other centers may further complement the conclusions.

### 4.3 Preoperative Plasma Fibrinogen, Monocyte‐Lymphocyte Ratio, and Erythrocyte Sedimentation Rate Predicted Postoperative Acute Infection

Our finding that plasma plasminogen, monocyte‐lymphocyte ratio, and erythrocyte sedimentation rate did not predict acute infection is similar to our previous finding that the three inflammatory markers were not associated with occult infection before total hip arthroplasty in patients with sequelae of suppurative hip arthritis [28]. However, our results contrast with a study in which plasma fibrinogen aided detection of periprosthetic joint infection before revision arthroplasty in patients without inflammatory diseases [29], highlighting the importance of studies specific to patients with rheumatoid arthritis. Another study with a relatively small sample reported that levels of plasma fibrinogen before revision surgery can aid the preoperative diagnosis of periprosthetic joint infection in patients with rheumatoid arthritis or other inflammatory diseases [30]. This finding does not necessarily contrast with ours given that we were trying to predict postoperative infection in a consecutive sample of patients, whereas that study was trying to diagnose infection preoperatively in patients who already had suspected infection.

## 5 Strengths and Limitations

The strength of our study is that this is the first time that inflammatory markers have been proposed before total joint arthroplasty for rheumatoid arthritis to predict the risk of postoperative infection, which could help reduce the occurrence of catastrophic complications. In addition, the data were collected from a large teaching hospital, and our perioperative management and surgical approach were standardized, which helped minimize the heterogeneity introduced by retrospective studies. Our findings should be interpreted with caution in light of its single‐center, retrospective design. Our retrospective study was designed to explore a potential association between postoperative acute infection and preoperative inflammatory markers, but not to the extent that a predictive model could be established. Therefore, a larger amount of data may be needed in the future to establish prediction models that are generalizable and stable. One source of selection bias in our sample may be that our center routinely checks C‐reactive protein and erythrocyte sedimentation rate in rheumatoid arthritis patients before surgery, and those with elevated values are referred to the rheumatology department to bring their disease activity under control before the operation. Furthermore, a single inflammatory marker may only increase sensitivity and decrease specificity. However, our sample size of patients with postoperative acute infections was small, and the use of two or more predictors may have biased the results. Therefore, it may be necessary to further explore the combination of two or more indicators to better predict the incidence of postoperative acute infection in samples with a larger sample size. In addition, our study did not discuss total joint arthroplasty in two subgroups: total knee arthroplasty and total hip arthroplasty. Given the relatively low incidence of infection, if the patients were further divided into two subgroups, too small sample size may lead to bias of the study results. Because of these limitations, our work should be verified and extended in larger, preferably multi‐center studies, especially to explore predictive cut‐off values.

### 5.1 Prospect of Clinical Application

The preoperative inflammatory indicators in patients with rheumatoid arthritis are higher than normal values, which makes the value of preoperative inflammatory indicators in predicting postoperative infection questioned. Our study initially suggests the use of NLR and optimal cutoff values to predict postoperative acute infection in patients with rheumatoid arthritis. If big data and machine learning methods can be combined in the future to develop more accurate preoperative inflammatory indicators and cut‐off values, it may be more accurate to predict the risk of postoperative acute infection, which may greatly reduce the risk of postoperative acute infection in patients with rheumatoid arthritis.

## 6 Conclusion

The preoperative NLR shows some ability to predict acute infection after total joint arthroplasty in patients with rheumatoid arthritis. Monitoring this ratio, perhaps in conjunction with other markers not analyzed here, may be useful for optimizing the timing of surgery in order to minimize risk of postoperative infection.

## Author Contributions

All authors had full access to the data of the study and take responsibility for the integrity and interpretation of the data. Conceptualization: Y.L., Z.L., and Z.Z. Methodology: Y.L. and J.F. Data curation: Y.L., X.L., N.L., and W.Z. Investigation: Y.L. and J.F. Software and formal analysis: N.L. and W.Z. Resources: Z.L. and Z.Z. Writing – original draft: Y.L. and J.F. Writing – review and editing: Y.L. Visualization: X.L. and Z.L. Project administration and supervision: Z.L. and Z.Z. All authors listed meet the authorship criteria according to the latest guidelines of the International Committee of Medical Journal Editors. All authors are in agreement with the manuscript.

## Ethics Statement

Approval for the study protocol was obtained from the Medical Research Ethics Committee at Sichuan University and the local village committee, aligning with the principles outlined in the Declaration of Helsinki. Prior to their participation in the study, all patients provided informed consent, underscoring the commitment to ethical standards and the protection of participants' rights.

## Conflicts of Interest

The authors declare no conflicts of interest.

## Data Availability

Data available on request from the authors.
